# Pennsylvania State Veterinary Medical Association

**Published:** 1901-07

**Authors:** 


### PENNSYLVANIA STATE VETERINARY MEDICAL ASSOCIATION.

Dr. H. P. Eves, of Delaware, was a visitor to the meeting, finding much to interest him along professional lines.

Dr. A. T. Sellers, of Camden, N. J., found time for his commercial duties to be in attendance at the meeting.

Not a section of the State was unrepresented in the convention, and the keenest interest shown among all who find this their yearly outing.

Dr. C. J. Marshall, as recording secretary, fills a very important post, and is a faithful servant of the Association.

